# Validation of a questionnaire designed to measure nursing student satisfaction with practical training

**DOI:** 10.1590/1518-8345.3102.3206

**Published:** 2019-12-05

**Authors:** Raimunda Montejano-Lozoya, Vicente Gea-Caballero, Isabel Miguel-Montoya, Raúl Juárez-Vela, Ángela Sanjuán-Quiles, Esperanza Ferrer-Ferrandiz

**Affiliations:** 1 Universidad de Valencia, Escuela de Enfermería La Fe, Valencia, Comunidad Valenciana, Spain.; 2 Instituto de Investigación Sanitaria IIS La Fe, Grupo de Investigación en Arte y Ciencia del Cuidado GREIACC, Valencia, Spain.; 3 Universidad de La Rioja, Facultad de Ciencias de la Salud, Logroño, La Rioja, Spain.; 4 Instituto de Investigación Sanitaria de Aragón IISA, Grupo de Investigación en Insuficiencia Cardiaca, Aragón, Spain.; 5 Universidad de Alicante, Departamento de Enfermería, Alicante, Comunidad Valenciana, Spain.; 6 Universidad de Alicante, Departamento de Enfermería, Grupo de Investigación: Salud Pública y Calidad de Vida, Bienestar Psicológico y Salud, Alicante, Spain.

**Keywords:** Personal Satisfaction, Education, Nursing, Nursing Education Research, Validation Studies, Surveys and Questionnaires, Students, Nursing, Satisfação Pessoal, Educação em Enfermagem, Pesquisa em Educação em Enfermagem, Estudos de Validação, Inquéritos e Questionários, Estudantes de Enfermagem, Satisfacción Personal, Educación en Enfermería, Investigación en Educación de Enfermería, Estudios de Validación, Encuestas y Cuestionarios, Estudiantes de Enfermería

## Abstract

**Objective:**

to validate an instrument designed to assess practical training and measure nursing student satisfaction with clinical practice modules.

**Method:**

cross-sectional study (academic year 2014-2015). Validation of the self-administered, anonymous, 17-item Nursing Student Satisfaction with Practical Training Questionnaire, developed by consensus by eight practical training experts in three nominal group sessions. Exploratory and confirmatory factor analyses were performed to assess internal consistency and validity. Student satisfaction was measured in relation to each module and all modules as a whole.

**Results:**

174 responses. High item-test correlation (≥0.90); Cronbach’s alpha = 0.91; Káiser-Meyer-Olkin index =0.86; the results of the Bartlett sphericity test were statistically significant (p<0.001); S-stress=0.17; R2=0.81. Exploratory factor analyses identified 4 factors: simulation, teacher tutoring, care facility selected for the placement, and clinician tutoring. Total explained variance was 66.6%. Confirmatory factor analysis obtained a chi-squared value of 285.275 (p= 0.000). Student satisfaction increases proportionately with each academic year.

**Conclusion:**

the questionnaire was shown to have good validity and is therefore a reliable instrument for measuring level of nursing student satisfaction with practical training in both clinical and simulated environments.

## Abstract (pt)

**Objetivo:**

validar um instrumento para avaliar a formação prática e a satisfação com as disciplinas de Prática Clínica de estudantes de enfermagem.

**Método:**

estudo observacional, descritivo, transversal (curso 2014-2015). Validação de um questionário autoadministrado e anônimo (17 itens) de satisfação em relação às disciplinas de Prática Clínica em estudantes de enfermagem, elaborado por consenso de oito especialistas em treinamento prático (em três sessões de grupo nominal). Foram realizadas análises fatorial e confirmatória, estimando-se a consistência interna para determinar a validade do instrumento. Foi calculada a satisfação dos alunos em cada disciplina e em sua totalidade.

**Resultados:**

174 respostas. Alta correlação entre itens (≥0,90); Alfa de Cronbach total=0,91; Kaiser-Meyer-Olkin=0,86; significância estatística no teste de esfericidade de Bartlett (p<0,001); S-stress=0,17; coeficiente de determinação R2=0,81. A análise fatorial exploratória encontrou 4 fatores: simulação, tutoria do professor, unidades assistenciais atribuídas e tutoria do profissional clínico. A variância total explicada foi de 66,6%. A análise confirmatória mostrou um qui-quadrado de 285,275 (p=0,000). A satisfação dos alunos avaliados aumentou proporcionalmente ao avanço do ano acadêmico.

**Conclusão:**

o Questionário de Satisfação sobre a Prática Clínica para Estudantes de Enfermagem atende aos requisitos de validade, sendo uma alternativa confiável para mensurar o grau de satisfação dos estudantes de enfermagem nos dois contextos das práticas clínicas atuais: ambientes clínicos e simulados.

## Abstract (es)

**Objetivo:**

validar un instrumento para valorar la formación práctica y la satisfacción con las asignaturas de Practicum de los estudiantes de Enfermería.

**Método:**

estudio observacional, descriptivo, transversal (curso 2014-2015). Validación de un cuestionario autoadministrado y anónimo (17 ítems) de Satisfacción sobre el Practicum en Estudiantes de Enfermería, elaborado por consenso de ocho expertos en formación práctica (en tres sesiones de grupo nominal). Se realizó análisis factorial y confirmatorio, estimándose la consistencia interna para determinar su validez. Se calculó la satisfacción de los estudiantes en cada asignatura y en su totalidad.

**Resultados:**

174 respuestas. Alta correlación entre ítems (≥0,90); alfa de Cronbach total=0,91; Káiser-Meyer-Olkin=0,86; significación estadística en prueba de esfericidad de Bartlett (p<0,001); S-stress=0,17; coeficiente de determinación R2=0,81. El análisis factorial exploratorio halló 4 factores: simulación, tutorización del profesor, unidad de cuidados asignada y tutorización del profesional clínico. Varianza total explicada=66,6%. El análisis confirmatorio mostró una Chi-square=285,275 (p= 0,000). La satisfacción de los estudiantes evaluados aumentó proporcionalmente según avanzaba el curso académico cursado.

**Conclusión:**

el cuestionario de Satisfacción de Practicum cumple los requisitos de validez, siendo una alternativa fiable para medir el grado de satisfacción de los estudiantes de Enfermería en los dos contextos de prácticas clínicas actuales: entornos clínicos y simulados.

## Introduction

One of the main challenges facing teachers and students in the field of health sciences is combining theoretical and practical knowledge, given the importance of the latter in the design of academic disciplines. Nursing science should incorporate practical nursing knowledge based on a rationale that draws on that which is already known and that which is acquired from practical experience. This requires a dialectic that combines reflection *in action, with action*, and *for action* played out in practical contexts marked by continuous decision-making to promote the development of judicious, critical, and reflective professionals^(^ 1 ^)^.

As other authors suggest, this reflection should be the bridge that unites what is set out in the academic curriculum and that which later constitutes professional development^(^ 2 ^-^ 3 ^)^. This learning, framed within a critically reflective perspective developed by the subjects who take part in the teaching and learning process, constitutes the central objective of new pedagogical approaches and a challenge that enables professionals to *create, change, redo, take risks, experiment, succeed, and fail* ^(^ 3 ^)^.

Royal Decree 861/2010, which governs Spanish higher education, brought the Spanish university system in line with the European Higher Education Area^(^ 4 ^)^. This new approach focused on learning how to learn and lifelong learning was a major turning point in nursing curriculums and degrees, separating and focusing learning in three distinct environments: the classroom (theoretical knowledge) and laboratory and clinical settings (practical knowledge). Strong emphasis is placed on supervised practice modules, developed in the second year with a minimum of 90 ECTS credits in compliance with the European Directive^(^ 5 ^-^ 7 ^)^.

The clinical learning process should enable students to develop reflective practice competencies that integrate the application of knowledge with skills and attitudes in real-life clinical situations and encourage them to reflect, internalize, and socialize professional values. At the same time, it should help students to identify components of the so-called “hidden curriculum” (meanings, characteristics and, in some cases, power relations)^(^ 8 ^)^. By caring for people during their practical training, students make sense of the theory learned in the classroom, contextualizing it in the time spent in care facilities and in the time spent studying and reflecting^(^ 9 ^)^.

Tutoring by clinicians is key to student learning. Tutors should be capable of taking students on a reflective journey through theoretical and practical knowledge^(^ 9 ^-^ 10 ^)^. They should also promote reflective debate in the classroom on students’ perceptions and problem-solving with the teachers responsible for the modules, encouraging reflective dialogue on actions undertaken in the care facilities. It is about stimulating students to investigate, search, and reflect on the practices learned.

There is no doubt that students undergo a process of evolution during the learning process which can often lead to stress; however, at the same time this process contributes to the transition that takes place between uncertainty in the first clinical practice modules and the sense of security students acquire towards the end of the course. A study with nursing students from a Spanish university showed that the greatest impact occurs in the realm of interpersonal relationships and behavior^(^ 11 ^)^.

In Spain, there are few validated instruments for measuring nursing student satisfaction with practical clinical training. We were unable to find an instrument that measures overall satisfaction with practical training in both real-life and simulated environments. A study that translated and validated the Clinical Learning Environment, Supervision and Nurse Teacher Evaluation Scale for use with Spanish students at the University of Alicante showed that the instrument had satisfactory psychometric properties for measuring student satisfaction in clinical environments, rather than simulated environments^(^ 12 ^)^. In terms of student satisfaction with practical training in simulated environments, we found a study conducted in Portugal that validated a questionnaire developed by the authors showing that the instrument had good validity and reliability^(^ 13 ^)^.

Given the importance of clinical practice modules for the student learning process and the lack of instruments for measuring student satisfaction, this study sought to develop and validate a questionnaire for measuring students’ opinions on practical training in both real-life and simulated environments. The main objective of the study was to assess the content and construct validity and reliability of the questionnaire, while the secondary objective was to determine the level of student satisfaction with the teaching methods implemented after the application of the Bologna agreements.

## Method

A cross-sectional study was conducted to assess the validity of the Nursing Student Satisfaction with Practical Training Questionnaire.

The nursing school where this study was conducted, *La Escuela de Enfermería de La Fe*, attached to the University of Valencia, established its Degree in Nursing in the academic year 2010-2011. The clinical practice modules fall within the core subject areas “Final Year Project”, with 7.5 credits, and “Integrated Practices in the La Fe Department of Health”, with a total of 84 credits, and are taken in the second, third, and fourth years of the course as follows: Year 1 - “Introduction to Nursing Practice”, with 6 credits; Year 2 - Clinical Practice I and Clinical Practice II, each with 19.5 credits; and Year 3 - Community Practice III with 19.5 credits and Clinical Practice IV in care facilities with 18 credits.

Each module consists of face-to-face study (80%) and non-face-to-face (autonomous learning) study (20%). Face-to-face study includes stays in health facilities, attendance at workshops, seminars, and learning activities tutored by teachers and clinicians^(^ 5 ^)^.

The study population was all students in the Degree in Nursing taking practice modules in the second, third, and fourth years during the study period.

The sample comprised students who accepted to participate in the study enrolled in the academic year 2014-2015 and taking practice modules conducted in a hospital setting, i.e. Introduction to Nursing Practice (second year), Clinical Practice II (third year) and Clinical Practice IV (fourth year).

The inclusion criteria were students enrolled in practice modules in the second, third, or fourth years of the degree program and students who wished to voluntarily participate in the study. The exclusion criteria were students enrolled in Clinical Practice I (the same students also enrolled in Clinical Practice II in the same academic year).

Sample size was calculated based on a minimum of 100 respondents^(^ 14 ^-^ 15 ^)^ and a minimum of 10 respondents per scale item^(^ 16 ^)^. The questionnaire was distributed to all students taking practice modules in a hospital setting.

The questionnaire was designed using the nominal group technique. During the first semester of the academic year 2014-2015, three sessions were held with a total of eight expert teachers belonging to the Department of Nursing at the University of Alicante and *La Escuela de Enfermería de La Fe*. The selection of experts was based on the following criteria: at least five years of experience of teaching or coordinating practices modules and experience of tutoring students in healthcare settings. The objective of these sessions was to reach a consensus on an instrument designed to assess level of student satisfaction with the practical training provided on the nursing degree program. To this end, the group studied the evaluation documents prepared by clinical tutors and teachers used by the two education centers.

After developing the draft instrument by consensus in the first session, the second and third sessions were used to define the questionnaire items based on the following content selection criteria: relevance, suitability, simplicity, and clarity of the proposed items. Finally, a pilot study was conducted to measure the internal consistency of the questionnaire using a sample of 53 students who had completed the module Clinical Practice II, resulting in a Cronbach’s alpha coefficient of 0.86, which is above the minimum acceptable value of 0.70 for determining test reliability^(^ 15 ^,^ 17 ^-^ 18 ^)^. The results therefore show high inter-item correlation and an acceptable level of internal consistency. No problems were reported by the study sample in relation to questionnaire wording and comprehension.

The final version of the questionnaire consisted of 17 items organized in thematic blocks: one comprising 10 items related to level of satisfaction with practice modules undertaken in care facilities and the other made up of seven items designed to measure level of satisfaction with learning in simulated clinical environments and theory-practice ratio. Each item was scored on a five-point Likert scale as follows: 1 = unsatisfactory, 2 = barely satisfactory, 3 = moderately satisfactory, 4 = satisfactory, and 5 = very satisfactory. The instrument was named the Nursing Student Satisfaction with Practical Training Questionnaire (*El Cuestionario de Satisfacción de Practicum en Estudiantes de Enfermería* - CSPEE).

Data was collected during the last week of each practice module. Each module coordinator provided students with a paper version of the questionnaire, together with instructions on how to return it to the academic secretary in a mailbox provided for this purpose.

The data was analyzed using descriptive statistics (frequencies and percentages) using the variables academic year and module. Mean and standard deviation was then calculated for each item. Internal consistency was measured using the item-test correlation, calculating Cronbach’s alpha for each deleted item and the 17 items as a whole using methodologies previously used by other authors^(^ 17 ^-^ 19 ^)^.

Exploratory factor analysis was preformed to identify the latent variables of the questionnaire, using principal component analysis and the Kaiser-Meyer-Olkin index and adopting a significance level of p<0.05. Confirmatory factor analysis was then performed to correct for possible inherent deficiencies of the exploratory factor analysis. This type of analysis provides more specificity for hypothesis testing. Confirmatory factor analysis was used to determine the validity of each item and identify common factors, verifying the contrast statistic of the hypothesis, as well as the analysis of covariance instead of correlation.

A nonparametric method was used for multidimensional scaling using the alternating least squares algorithm. Finally, k-means clustering was used to determine satisfaction in each of the factors.

The Bonferroni test was performed, which allows multiple comparisons to be made to determine student satisfaction across the three practice modules studied.

Statistical analysis was performed using the statistical software the Statistical Package for the Social Sciences (version 20 for Windows), adopting a 95% confidence interval (CI).

The study was authorized by the Board of Directors of the *Escuela de Enfermería de La Fe* and the school’s Ethics Committee. All participants signed an informed consent form and the questionnaires were filled out anonymously, protecting the identity of the respondents and the practice tutors and health facilities being evaluated. The data was processed in accordance with the European Union’s General Data Protection Regulation 2016/679, applied in Spain on May 25, 2018^(^ 20 ^)^.

## Results

From a total of 191 students enrolled in practice study modules in the second, third, and fourth years of the Degree in Nursing in the academic year 2014-2015, 174 responded the questionnaire (response rate = 91%). Of this group, 63 (36.2 %) had taken the module Introduction to Nursing Practice, 53 (30.5 %) Clinical Practice II, and 58 (33.3 %) Clinical Practice IV.

First we determined whether the items of the CSPEE were related to satisfaction with practical training using Cronbach’s alpha, resulting in a value of ≥0.90 for all items (Table 1).

**Table 1:** Item homogeneity and internal consistency of the CSPEE measured with Cronbach's alpha (n=174). Valencia, Spain, 2014-2015

Item	Mean	SD*	Corrected item-total correlation	Alpha if item deleted
Teacher tutoring process	3.71	1.14	.69	.90
Clinician tutoring process	3.73	1.14	.66	.90
Proposed tutoring method	3.19	.99	.50	.90
Help received from the teacher	3.66	1.17	.69	.90
Help received from the clinician	3.66	1.13	.67	.90
Collaboration of other professionals in learning	4.09	.97	.29	.91
Practice module duration	3.30	1.16	.41	.90
Health facility assigned for acquisition of clinical competencies	4.1	.95	.27	.91
Overall level of satisfaction with the practice modules	4.13	.93	.53	.90
Practice module monitoring and evaluation process was adequate	3.44	1.01	.57	.90
Level of previous knowledge prior to doing the practice modules	3.32	.86	.36	.90
Information received (theoretical/practical-laboratory content, simulation…)	3.47	1.05	.55	.90
Methods used by teachers in the weekly clinical sessions (presentation, audiovisual media, review of care plan, material…)	3.14	1.0	.60	.90
Teacher's knowledge of the topics and clarity of explanation	3.28	.99	.56	.90
Duration of clinical sessions (presentations and practices) was adequate	2.97	1.09	.57	.90
Organization and planning of clinical sessions/module monitoring and control	2.95	1.08	67	.90
Usefulness of the skills acquired during practical training, laboratory, simulation, problem-based learning, etc. for clinical practice	3.55	1.02	.51	.90

* SD = Standard deviation

Construct validity was measured using exploratory factor analysis to identify the latent variables of the questionnaire by performing principal module analysis, resulting in a Kaiser-Meyer-Olkin index of 0.86. The results of Bartlett’s test of sphericity were statistically significant (p<0001), obtaining a chi-square value of 1473.9.

The results of the nonparametric method used for multidimensional scaling using the alternating least squares algorithm were as follows: for the matrix: S-stress = 0.1675; and coefficient of determination (R^2^) = 0.80597.

Principal component analysis was followed by the application of varimax rotation to reduce the number of variables with high factor loadings. Four factors were selected and total variance and the percentage of variance explained by each factor was calculated. Factor 1 (simulation), comprising 6 items (12,13,14,15,16,17) related to the dimension practice in “simulation”, explained 41.6% of total variance; factor 2, consisting of 4 items (1,3,4,10) related to “teacher tutoring process”, explained 10.1% of total variance; factor 3, composed of 4 items (7,8,9,11) related to the “selected health facility”, explained 8.3% of total variance; and factor 4, made up of 3 items (2,5,6) related to the “clinician tutoring process”, explained 6.6% of total variance (Table 2). Total explained variance was 66.6%.

**Table 2:** Rotated component matrix: 4-factor Varimax with Kaiser Normalization. Valencia, Spain, 2014-2015

Item	Factor 1	Factor 2	Factor 3	Factor 4
Organization and planning of clinical sessions/module monitoring and control	.789			
Methods used by teachers in the weekly clinical sessions (presentation, audiovisual media, review of care plan, material…)	.785			
Duration of clinical sessions (presentations and practices) was adequate	.785			
Teacher's knowledge of the topics and clarity of explanation	.704			
Information received (theoretical/practical-laboratory content, simulation…)	.609			
Usefulness of the skills acquired during practical training, laboratory, simulation, problem-based learning, etc. for clinical practice	.580			
Teacher tutoring process		.865		
Help received from the teacher		.851		
Practice module monitoring and evaluation process was adequate		.695		
Proposed tutoring method		.625		
Health facility assigned for acquisition of clinical competencies			.706	
Overall level of satisfaction with the practice modules			.703	
Practice module duration			.481	
Level of previous knowledge prior to doing the practice module			.380	
Help received from the clinician				.876
Clinician tutoring process				.835
Collaboration of other professionals in learning				.544

The results of the confirmatory factor analysis were as follows: Chi-squared=285.275 (df=113), p= 0.000; comparative fit index (CFI) =.877; normative fit index (NFI)=.814; root-mean-square error of approximation (RMSEA) =.094. Various goodness of fit tests were used to test the model, including the chi-squared test (χ2), comparative fit index (CFI), normative fit index (NFI), and mean-square error of approximation (RMSEA). A CFI of > 0.90 indicates acceptable fit of the model to the data. A RMSEA of <0.05 suggests a good model fit. NFI values of close to 1.0 are preferred according to some authors^(^ 21 ^-^ 22 ^)^.

**Figure 1:**
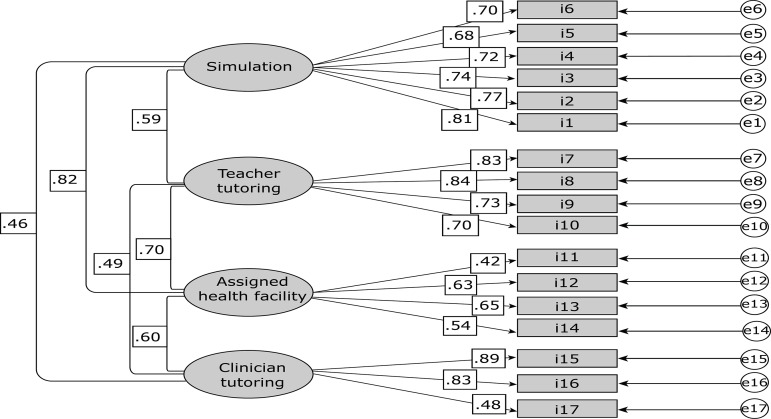
Flow diagram*. Valencia, Spain, 2014-2015 *The rectangles represent the items. The ellipses represent the common factors. The one-direction arrows between the common factors and items express saturation. The two-direction arrows indicate a correlation between common and unique factors

Based on the recommendations of several authors^(^ 15 ^,^ 23 ^-^ 24 ^)^, for the model to be accepted the p-value should be > 0.05, since the null hypothesis states that the model is not significant (Figure 1).

K-means clustering was used to measure satisfaction for each dimension of the questionnaire. Table 3 shows that satisfaction was over 59% across all dimensions, with the dimension related to the health facility assigned for acquisition of clinical competencies obtaining the highest percentage (64.9%) and the dimension related to clinician tutoring the lowest (59.8%)

**Table 3:** Satisfaction by factor. Valencia, Spain, 2014-2015

Factor		n	%
Factor 1 (6 items) Simulation	No	65	37.4
	Yes	109	62.6
Factor 2 (4 items) Teacher tutoring	No	69	39.7
	Yes	105	60.3
Factor 3 (4 items) Care facility	No	61	35.1
	Yes	113	64.9
Factor 4 (3 items) Clinician tutoring	No	70	40.2
	Yes	104	59.8

The results obtained in the Bonferroni test were based on bilateral tests assuming equal variances with a significance level of 0.05. For each significant pair, the key of the category with the smaller mean was placed under the category with larger mean.

The tests were adjusted for all pairwise comparisons within a row for each innermost sub-table using Bonferroni correction. The results are shown in Figure 2.

**Figure 2:** Satisfaction with the modules. Valencia, Spain, 2014-2015

Item		Module I (2nd year) A	Module II (3rd year) B	Module IV (4th year) C
**1**	Teacher tutoring process			A
**2**	Clinician tutoring process			A B
**3**	Proposed tutoring method		A	A B
**4**	Help received from the teacher			A
**5**	Help received from the clinician			A B
**6**	Collaboration of other professionals in learning			A
**7**	Practice module duration		A	A B
**8**	Health facility assigned for acquisition of clinical competencies			B
**9**	Overall level of satisfaction with the practice modules		A	A
**10**	Practice module monitoring and evaluation process was adequate			A
**11**	Level of previous knowledge prior to doing the practice module			A
**12**	Information received (theoretical/practical-laboratory content, simulation…)			A B
**13**	Methods used by teachers in the weekly clinical sessions (presentation, audiovisual media, review of care plan, material…)			A B
**14**	Teacher's knowledge of the topics and clarity of explanation			A B
**15**	Duration of clinical sessions (presentations and practices) was adequate			A B
**16**	Organization and planning of clinical sessions/module monitoring and control			A B
**17**	Usefulness of the skills acquired during practical training, laboratory, simulation, problem-based learning, etc. for clinical practice		A	A

Significant differences (p<0.05) were found between the modules Introduction to Nursing Practice (second year) and Clinical Practice II (third year) for the following items: teaching tutoring process, practice module duration, overall satisfaction with the practice modules, and usefulness of the skills acquired for clinical practice. The results show that third year students obtained higher scores. This difference was shown to be statistically significant (p<0.05).

The comparison of the modules Introduction to Nursing Practice (second year) and Clinical Practice IV (fourth year) showed that all items except the health facility assigned for acquisition of clinical competencies showed higher scores in Clinical Practice IV. The difference between the means of the modules was statistically significant (p<0.05).

The comparison of the modules Clinical Practice II (third year) and Clinical Practice IV (fourth year) showed that scores were higher in Clinical Practice IV for the following items: clinician tutoring process, proposed tutoring method, help received from the clinician, practice module duration, health facility assigned for acquisition of clinical competencies, overall satisfaction, information received, methods used by teachers in the weekly clinical sessions, teacher’s knowledge of the topics and clarity of explanation, duration of clinical sessions, and organization and planning of clinical sessions. These differences were statistically significant (p<0.05).

## Discussion

This study attempted to propose a questionnaire that provides a valid measure of nursing student satisfaction across all dimensions of their practical training in both simulated and clinical environments. This part of the learning process is vital for nursing students, since it brings together previously acquired knowledge and a deeper applied knowledge of the discipline, laying the foundation for meaningful learning by encouraging reflection in action^(^ 1 ^-^ 3 ^)^. Hence the importance of developing ways and instruments that enable the accurate measurement of the practical training process as a whole, encompassing both real-life and simulated environments.

Previously validated instruments have been used to assess practical training in clinical and simulated environments separately^(^ 12 ^-^ 13 ^)^. With the development and validation of this questionnaire it is now possible to measure training as a single, inseparable process, thus avoiding separate measures with different instruments, which, despite being acceptable, are often developed in different contexts and have unequal psychometric properties. The fact that it is possible to measure both learning environments using a single instrument is one of the strengths of this study.

Student growth and development promotes progress and the transition to professional practice and therefore strategies designed to teach practical skills should be developed as a single, natural, progressive, and inseparable process. As such, these strategies should be measured as a whole, since it is student progress that enables the development of both the formal and the so-called hidden curriculum^(^ 8 ^)^. The practical training process is vital for student maturation as it provides knowledge and experience of the idiosyncratic aspects of the profession, social and healthcare settings, human relations, their relationship with other professions, and interdisciplinary teamwork.

With regard to item validity and questionnaire reliability, the value for Cronbach’s alpha was high (0.91), suggesting that all the items are related to satisfaction with the practical training process. The fact that all items obtained an alpha coefficient of ≥0.90 and that the elimination of any item would jeopardize the scale shows that the instrument has good construct validity and adequately measures the intended construct^(^ 15 ^,^ 24 ^-^ 25 ^)^. Given that we measured construct validity, before applying factor analysis to the data the Bartlett sphericity test was performed to show whether the sample number was sufficient to conduct the analysis. The values obtained (chi-squared=1473.9 for a p-value of <0.001) showed that the sample size was suitable for factor analysis. Moreover, the results of the Kaiser-Meyer-Olkin test (0.86), also performed before factor analysis, confirmed sampling adequacy.

The values obtained from multidimensional scaling (<0.2, S-stress=0.1675) show that the algorithm was programmed correctly^(^ 24 ^,^ 26 ^)^. Furthermore, the R^2^ value was close to 1 (0.80597), indicating adequate spread and that the proportion of the spread of theoretical values in our study is 80.6%.

After defining the four factors using Varimax rotation with Kaiser Normalization (to reduce the number of variables with high factor loadings), we calculated total variance and the percentage of variance explained by each factor, obtaining good results for total explained variance (66.6%) and for each dimension, particularly factor 1 (simulation), which explained 41.6% of total variance.

With respect to the questionnaire results, the scores show that student satisfaction with clinical placements increases proportionately with academic year. This finding is particularly relevant since the training program is designed to promote the progressive immersion of students in nursing practice, while at the same time gradually fostering reflective processes that combine theoretical and practical knowledge. The maximum level of student satisfaction occurs in the fourth and final year, when student practice most closely resembles professional practice. These findings are in line with those of a previous study^(^ 11 ^)^, confirming that nursing student learning is progressive, involving a gradual transition from uncertainty in the first practice modules to a sense of security in the final stages of the course, which supports our consideration regarding the evolution of student satisfaction.

The findings also show that the level of satisfaction was high for all dimensions, most notably for the health facility assigned for acquisition of clinical competencies. Satisfaction percentages of over 59% show that the majority of students are satisfied, both overall and for each dimension of the questionnaire. It is interesting to note that, although relatively high (59.8%), the dimension that obtained the lowest level of satisfaction was clinician tutoring. This dimension shows room for improvement, given that other authors have reported that students view tutoring by clinicians as a vital component of their training^(^ 9 ^,^ 12 ^,^ 26 ^-^ 27 ^)^.

The main contribution of this study to the body of knowledge in this area is that it provides a validated tool for evaluating clinical learning processes in dual environments (real-life and simulated), thus filling a current gap, given the lack of validated instruments adapted to evaluate practice modules in courses in mixed environments in the field of health sciences.

One of the limitations of this study is that our findings do not confirm the validity and robustness of the CSPEE for measuring satisfaction with practical training in other health facilities and other disciplines in the field of health sciences. In this respect, further research is needed to assess the validity of this instrument for courses in areas such as medicine, physical therapy, chiropody, and dentistry.

## Conclusion

The main contribution of this study is that it was able to integrate the measurement of student satisfaction with practical training in both real-life and simulated environments into the same questionnaire. The instrument was shown to have satisfactory psychometric properties, confirming that it adequately measures the intended construct. The Nursing Student Satisfaction with Practical Training Questionnaire is therefore a reliable instrument for measuring nursing student satisfaction with practical training as a whole, including both real-life and simulated learning environments.
